# Treatment switch from multiple daily insulin injections to sulphonylureas in an African young adult diagnosed with *HNF1A* MODY: a case report

**DOI:** 10.1186/s13256-024-04850-3

**Published:** 2024-10-17

**Authors:** Jean Claude Katte, Mesmin Y. Dehayem, Kevin Colclough, Eugene Sobngwi

**Affiliations:** 1 Department of Clinical and Biomedical Science, Faculty of Life and Health Science, University of Exeter Medical School, Exeter, UK; 2 National Obesity Centre and the Endocrinology and Metabolic Diseases Unit, Yaoundé Central Hospital, Yaoundé, Cameroon; 3 Department of Non-Communicable Diseases, RSD Institute, Yaoundé, Cameroon; 4 Department of Internal Medicine and Specialities, Faculty of Medicine and Biomedical Sciences, University of Yaoundé 1, Yaoundé, Cameroon; 5 Exeter Genomics Laboratory, Royal Devon University Healthcare NHS Foundation Trust, Exeter, UK

**Keywords:** *HNF1A* MODY, Type 1 diabetes, Diabetes, Insulin, Cameroon, Sub-Saharan Africa

## Abstract

**Background:**

Maturity onset diabetes of the young is one of the commonest causes of monogenic diabetes and can easily be mistaken for type 1 diabetes. A diagnosis of maturity onset diabetes of the young can have direct implications for genetic counseling, family screening, and precision diabetes treatment. However, the cost of genetic testing and identifying individuals to test are the main challenges for diagnosis and management in sub-Saharan Africa. We report the very first documented case of HNF1A maturity onset diabetes of the young in the sub-Saharan African region.

**Case presentation:**

A 20-year-old female Black African young adult diagnosed with type 1 diabetes aged 14 presented for routine out-patient diabetes consultation. She was on multiple daily insulin injections; total combined dose 0.79 IU/kg/day with an HbA1c of 7.7%. The rest of her laboratory examinations were normal. On extended laboratory analysis, she had good residual insulin secretion with post-meal plasma C-peptide levels at 1150 pmol/L. She tested negative for glutamic acid decarboxylase (GAD65), islet antigen-2 (IA-2), and zinc transporter 8 (ZnT8) islet autoantibodies. Targeted next-generation sequencing (t-NGS) for monogenic diabetes was performed using DNA extracted from a buccal sample. She was diagnosed with *HNF1A* maturity onset diabetes of the young, with the c.607C > T; p.(Arg203Cys) pathogenic variant, which has never been reported in sub-Saharan Africa. Her clinical practitioners provided genetic and therapeutic counseling. Within 10 months following the diagnosis of maturity onset diabetes of the young, she was successfully switched from multiple daily insulin injections to oral antidiabetic tablets (sulphonylurea) while maintaining stable glycemic control (HBA1c of 7.0%) and reducing hypoglycemia. She expressed a huge relief from the daily finger pricks for blood glucose monitoring.

**Conclusion:**

This case reveals that *HNF1A* maturity onset diabetes of the young (and probably other causes of monogenic diabetes) can present in sub-Saharan Africa. A diagnosis of maturity onset diabetes of the young can have significant life-changing therapeutic implications.

## Background

Maturity onset diabetes of the young (MODY) is the commonest form of monogenic diabetes, a rare group of heterogeneous endocrine disorders resulting from the mutations of genes critical to the development and/or function of the pancreatic beta cells. MODY can easily be mistaken for either type 1 or type 2 diabetes. Classical clinical diagnostic features for recognizing MODY are young age at diabetes onset (< 25 years), family history of diabetes in a parent, evidence of residual insulin secretion, and the absence of islet cell autoimmunity [1, 2]. However, the definitive diagnosis of MODY can only be confirmed by genetic testing using molecular techniques to identify mutations [3]. Making the correct diagnosis of MODY is critical for diabetes precision medicine [4]. For example, the correct diagnosis of MODY can prompt diabetes treatment cessation or the discontinuation of insulin depending on the MODY genes and clinical profile of the patients [5].

The burden and the clinical benefits of making a diagnosis of MODY in continental black African populations are largely unknown [6]. This can mainly be attributable to the relatively expensive molecular methods of diagnosis (even for developed countries) but also due to a low index of suspicion in African clinical settings [6–8]. Identifying individuals with MODY in Africa may be helpful to correctly selecting individuals who may transition from over-treatment with insulin (which is expensive, requiring multiple daily injections and glucose monitoring) to cheap oral antidiabetic tablets that are readily available, affordable, easy to administer and generally accepted by most patients. In this report, we present the case of a diagnosis of *HNF1A* MODY in a black sub-Saharan African young adult previously diagnosed with type 1 diabetes and treated with multiple daily insulin injections. To the best of our knowledge, this is the very first documented case of *HNF1A* MODY in the sub-Saharan African region. The diagnosis of MODY led to insulin cessation with transition to oral antidiabetic treatment with sulphonylurea while maintaining optimal glucose control.

## Case presentation

A 20-year-old female Black African young adult presented at the out-patient department for routine consultation and follow-up. She was previously diagnosed with type 1 diabetes at the age of 14 years following presenting with an abrupt onset of osmotic symptoms (thirst, polyuria, and unexplained weight loss), random plasma glucose at 471 mg/dL (25.9 mmol/L), and ketonuria at 8.0 mmol/L. Her weight at the time of diabetes diagnosis was 50 kg, and HbA1c was at 10.9%. She is an only child and has no known parental history of diabetes. Her father was deceased, and whether he had diabetes cannot be ascertained. She was immediately hospitalized, and ketosis was corrected with intravenous fluids and insulin per standard protocol. Basic education about type 1 diabetes was provided to the patient and her mother. She was discharged after 1 week on daily multiple insulin injections. Her insulin treatment (including accessories such as insulin syringes, glucose meter, and test strips) was provided free of charge through the Changing Diabetes in Children (CDiC) program in Cameroon. The CDiC program provides free comprehensive care to children and young adults diagnosed with childhood diabetes [9].

At clinic review on the day of presentation, she reported never having been hospitalized neither for diabetes ketoacidosis (DKA) nor severe hypoglycemia since her diagnosis. She admits having some intermittent episodes of hypoglycemia (nonquantifiable) within the last 6 months, which were corrected by eating. She demonstrated knowing the signs and symptoms of hypoglycemia, and what to do to correct a mild event. Her insulin regimen was Actrapid® 18 IU in the morning, 18 IU at lunch time, and 18 IU of Mixtard 30® before her evening meal. A total dose of 54 IU/day representing 1.08 U/kg/day. She reported monitoring her blood glucose on average seven times in a week. Her vital signs were normal, weight and height at 58 kg and 170 cm, respectively [calculated body mass index (BMI) at 20.0 kg/m^2^]. She reported having a regular 28-day menstrual cycle with a menarche at 12 years. Her injection sites, feet, and the rest of the physical examination was unremarkable. There were no signs of distal peripheral neuropathy upon clinical examination, and she reported never having been diagnosed with any associated autoimmune or chronic medical condition. Urine analysis showed heavy glycosuria (60 mml/L) without significant ketonuria or proteinuria. She reported living alone as an undergraduate university student and would usually use public transportation to attend her clinic appointments. She does not have a medical insurance and described herself as being in an excellent state of health. She did not look distressed and reported not being stigmatized because of her diabetes.

Her laboratory analysis showed an HbA1c was 7.7% with evidence of considerable endogenous insulin secretion with a stimulated plasma C-peptide of 1150 pmol/L. Plasma C-peptide was measured within 5 hours of her routine home meal in the nonfasting state (nonfasting C-peptide measurement). The serological analysis for islet autoantibodies were all negative; glutamic acid decarboxylase (GAD65, < 5.0 U/mL), insulin antigen 2 (IA-2, < 7.5 U/mL), and zinc transporter 8 (ZnT8, < 10 U/mL). Lipids and liver function were normal. The rest of the laboratory examination results are summarized in Table 1.

**Table 1:** Results of the extended laboratory analysis from peripheral venous blood and urine collected on the day of consultation

Blood or urine parameters	Results	Interpretation
*Peripheral blood*		
White blood cells (/µL)	4400	Normal
Hemoglobin (g/dL)	14.6	Normal
Hematocrit (%)	42.6	Normal
Platelets (/µL)	206,000	Normal
*Blood biochemistry*		
Albumin, (g/L)	52	Normal
Aspartate aminotransferase (AST), (U/L)	12	Normal
Alanine aminotransferase (ALT), (U/L)	8	Normal
Alanine phosphatase, (U/L)	99	Normal
Total bilirubin, (µmol/L)	9	Normal
Creatinine, (µmol/L)	77	Normal
Total cholesterol, (mmol/L)	4.7	Normal
High-density lipoprotein cholesterol, (mmol/L)	1.68	Normal
Triglycerides, (mmol/L)	0.96	Normal
Plasma glucose, (mg/dL/mmol/L)	167/9.2	Elevated
Glycated hemoglobin, (%)	7.7	Elevated
Plasma C-peptide, (pmol/L)	1150	Elevated
*Immunological markers*		
Glutamic acid decarboxylase (GAD), (U/mL)	< 5.0	Negative
Insulin antigen 2 (IA-2), (U/mL)	7.5	Negative
Zinc transporter 8 (ZnT8), (U/mL)	< 10	Negative
Tyroxine peroxidase (mIU/L)	9	Normal
Tissue transglutaminase, (IU/mL)	0.4	Normal
*Urine dipstick tests*		
pH	7.0	
Glucose, mmol/L	60	Glycosuria
Ketone, mmol/L	1.5	Normal
Protein, mg/dL	30	Normal

Based on the elevated plasma C-peptide levels 6 years post diagnosis and negative islet autoantibody (GAD65, IA-2, ZnT8) tests, relatively good glycemic control, and the absence of any chronic medical condition, she was recommended for monogenic diabetes testing. Pretesting counseling was provided by a specialized nurse before sample collection. Saliva samples were collected in a Isohelix GeneFix® Saliva DNA collection tube and sent to the Exeter Genomics Laboratory for monogenic diabetes testing about 15 months after the initial assessment. A total of 57 previously described monogenic diabetes genes were tested using a targeted next-generation sequence (t-NGS) panel at the Exeter Genomics Laboratory of the Royal Devon University Healthcare NHS Foundation Trust, Exeter, UK [3]. A diagnosis of hepatocyte nuclear factor 1 alpha MODY (*HNF1A* MODY) was confirmed due to a known pathogenic heterozygous missense mutation c.607C > T and protein effect p.(Arg203Cys) [10].

Upon the return of the molecular diagnosis of MODY, she was invited for genetic counseling on the implications of the diagnosis, importance of family screening, and the possibility of switching to an oral antidiabetic treatment. Her mother was invited for counseling and testing (HbA1c 4.5% and saliva was collected for future testing). At this consultation, the patient reported several hypoglycemic episodes that were self-corrected. Her random blood glucose was 114 mg/dL (6.2 mmol/L) and HbA1c at clinic was at 6.2%. Her insulin treatment was reduced to two injections of NPH (neutral protamine hagedorn) insulin at 12 IU in the morning and 12 IU in the evening before meals. After 1 week of treatment, she reported three episodes of nonsevere hypoglycemia. Her insulin was further reduced to 8 IU of NPH insulin morning and evening before meals and glimepiride 2 mg was started one tablet per day in the morning. She later reported having a severe hypoglycemic episode and was advised to stop insulin injections and continue with glimepiride 2 mg twice daily. Three months later, after transitioning from multiple daily insulin injections to sulphonylurea tablets (glimepiride), her HbA1c was at 5%. She has not reported any hypoglycemic episodes since switching to sulphonylurea treatment alone. Also, she has not reported any other adverse effect or intolerance to her new treatment. On routine consultation, 1 year after insulin cessation and the transitioning to glimepiride tablets, her weight is 55 kg (BMI 19.0 kg/m^2^) and HbA1c 7.0%. Based on her medical records, she has not been diagnosed with any chronic medical condition such as diabetic retinopathy, peripheral neuropathy, or any cardiovascular or severe neurological condition. She reported being relieved from the daily finger pricks from capillary blood glucose monitoring she was accustomed to under her previous treatment regimen. Her long-term diabetes management plan as noted in her medical record was to continue her sulphonylurea treatment while monitoring HbA1c at least twice a year, and to intensify therapy if necessary.

## Discussion

We have reported on a case of *HNF1A* MODY in a female young adult living in a sub-Saharan African setting, who was misdiagnosed as having type 1 diabetes, and treated with multiple insulin injections. The correct diagnosis of *HNF1A* MODY with adequate therapeutic counseling led to insulin cessation and a transition to oral antidiabetic treatment with sulphonylurea while maintaining optimal glycemic control.

To the best of our knowledge, this is the first case report on *HNF1A* MODY in a sub-Saharan African setting, clearly highlighting the clinical benefits of making this diagnosis. The burden of MODY and other forms of monogenic diabetes is currently unknown in sub-Saharan Africa. This may be attributable to the low clinical index of suspicion among clinicians but mostly probably due to the high cost of genetic testing. There are currently only two reported cases of permanent neonatal diabetes mellitus (a form of monogenic diabetes usually diagnosed in infancy) in sub-Saharan Africa. The first reported case was that of a Ugandan male toddler (approximately 19 months old at the time of diagnosis) with a missense mutation p.(Arg201Cys) in the *KCNJ11* gene [11]. The second reported case was a Togolese 8 year old female child diagnosed with a novel heterozygous missense mutation p.(Phe333Val) in the *KCNJ11* gene [12]. The diagnosis of these cases was confirmed using Sanger sequencing at the Exeter Genomics Laboratory, which serves as a UK national reference center for monogenic diabetes genetic testing, but also provides services throughout the globe. There are multiple reports of MODY and other syndromic monogenic diabetes forms in North African populations [13–15]. This may be as a result of a high index of clinical suspicion informed by the predominance of other genetic diseases from the high rates of consanguineous marriages [16]. Also, accessibility to molecular testing techniques either within Northern African countries or due to their relative proximity to Europe can provide an added advantage to this region compared with sub-Saharan Africa.

To date, mutations in 11 genes have been reported to cause MODY [2]. The majority of MODY cases result from mutations in the *GCK* genes encoding glucokinase (*GCK* MODY) and the hepatocyte nuclear factor genes *HNF1A* and *HNF4A* [17]. Our patient was diagnosed with *HNF1A* MODY. *HNF1A* mutations result in progressive beta cell decline characterized by reduced beta cell proliferation and increased beta cell apoptosis. Clinically, patients with HNF1A mutations will manifest glucose intolerance during adolescence or early adulthood with progressive hyperglycemia, and low renal glucose threshold. This is consistent with the diagnosis of diabetes at 14 years in our case. *HNF1A* MODY is known to be extremely sensitive to sulphonylureas [1, 2, 18]. Improved glycemic control with sulphonylurea treatment has been demonstrated in individuals with shorter durations of diabetes and lower HbA1c and BMI at the time of transition to insulin [19, 20]. This may explain our patient's good glycemic control even after treatment switch from insulin to sulphonylurea treatment given her relatively shorter diabetes duration, low BMI, and residual endogenous insulin secretion. This treatment change can be of great significance in sub-Saharan Africa where access to and adherence to insulin treatment can be extremely challenging [21]. Furthermore, sulphonylurea regimens are easy to administer, cheap, readily available in most low- and middle-income countries (LMICs), and do not require frequent monitoring of blood glucose levels [22].

Our patient reported no family history of diabetes. Her only living parent (mother) is reported not to have diabetes (HbA1c at 4.5%). Genetic testing of her mother’s saliva sample was negative for *HNF1A* MODY. We do not know if her deceased parent (father) had diabetes. She has no other siblings and currently has no child of her own. It is also possible that this may be a case of a spontaneous de novo *HNF1A* mutation, as they have been demonstrated in other populations [17]. Generally, mutations in *HNF1A* have demonstrated high penetrance in European populations, but studies are lacking in African populations.

Typically, MODY is usually diagnosed in adolescence (usually before 30 years), in individuals with a parental history of diabetes, who are nonobese and do not present with signs of insulin resistance or dyslipidemia. MODY can be very clinically heterogeneous depending on the specificity of the genetic mutation [23]. The patient in this case report was previously diagnosed with type 1 diabetes based on her young age at diagnosis (14 years), acute presentation, with some evidence of glucotoxicity and ketosis. MODY is often misdiagnosed as type 1 or type 2 diabetes in most clinical settings given that the signs and symptoms at diagnosis overlap [2]. Extended laboratory analysis showed she was islet autoantibody negative and C-peptide positive (C-peptide > 200 pmol/L). The good residual endogenous insulin secretion (post-meal C-peptide level: 1150 pmol/L) 6 years after the diagnosis of type 1 diabetes was the main indicator prompting genetic testing for monogenic diabetes. Other useful prognostic characteristics that could have been useful towards the clinical suspicion of MODY were the relatively good glycemic control and absence of chronic complications, although these are very nonspecific [23]. The selection of individuals for monogenic diabetes genetic testing based on a biomarker approach (including C-peptide and islet autoantibodies) has demonstrated to provide an improvement in yield than the classical clinical approach [24]. Therefore, this biomarker selection approach may prove useful in the future to establishing the burden of MODY in African populations given it has been demonstrated to target those most likely to have a positive monogenic diabetes test result [23, 24].

This case report clearly underscores the life-changing implications a single diagnosis of MODY can bring to the life of a person living with diabetes. Making the diagnosis of MODY in this case was challenging given the circumstances. There are currently, to the best of our knowledge, no routine genetic testing centers for monogenic diabetes in Cameroon or any other sub-Saharan Africa country. With funding made available through a research grant, we were able to collect, ship and analyze her saliva sample. The saliva sample was collected using an Isohelix GeneFix® Saliva DNA collection tube (www.isohelix.com). The Isohelix collection tube offers long-term sample stability at normal ambient temperature, without additional storage cost as opposed to blood samples. The diagnosis was made some 15 months after the initial review owing to constrains around arranging for shipment and analysis.

There were some limitations. We were unable to capture relevant information regarding her dietary habits and other lifestyle components such as physical activity over the course of her follow-up. We did not collect data regarding adherence to either insulin or the sulphonylurea treatment, although she reported being adherent to her treatment as prescribed by the doctors. Lastly, we did not objectively measure quality of life (QoL) indices before and after the treatment switch. However, she reported a huge relief from the numerous finger-pricks for self-monitoring of blood glucose (SMBG).

## Conclusion

This case reveals that *HNF1A* MODY (and probably other causes of monogenic diabetes) can present in sub-Saharan Africa, just like in other parts of the world, and a diagnosis of MODY can lead to significant life-changing therapeutic implications for the patient.

## Data Availability

All the data for this case report have been included in this article. Further enquiries can be provided upon reasonable request from the corresponding author.

## Abbreviations

CDiC: Changing diabetes in children
HbA1c: Glycated hemoglobin
HNF1A: Hepatotcyte nuclear factor 1 alpha
LMICs: Low- and middle-income countries
MODY: Maturity onset diabetes of the young
NGS: Next-generation sequencing
SMBG: Self-monitoring of blood glucose
SSA: Sub-Saharan Africa
QoL: Quality of Life
